# Protective effect of short-chain fructo-oligosaccharides from chicory on alcohol-induced injury in GES-1 cells via Keap1/Nrf2 and NLRP3 inflammasome signaling pathways

**DOI:** 10.3389/fnut.2024.1374579

**Published:** 2024-05-14

**Authors:** Yan Chen, Yanan Zhao, Hao Lu, Weichen Zhang, Yanan Gai, Guanting Niu, Xiuhua Meng, Han Lv, Xiaoguo Qian, Xiaoqin Ding, Jian Chen

**Affiliations:** ^1^Jiangsu Key Laboratory for the Research and Utilization of Plant Resources, Institute of Botany, Jiangsu Province and Chinese Academy of Sciences, Nanjing, China; ^2^School of Pharmacy, Nanjing University of Chinese Medicine, Nanjing, China; ^3^Fengning PingAn High-Tech Industrial Co., Ltd, Chengde, China

**Keywords:** scFOS, chicory plant, gastric cell damage, oxidative stress, Keap1/Nrf2 signaling, NLRP3 inflammasome signaling

## Abstract

Numerous studies have demonstrated that polysaccharides derived from chicory possess the ability to regulate host signaling and modify mucosal damage. Yet, the effect and mechanism of short-chain fructo-oligosaccharides (scFOS) on gastric mucosa remain unclear. Hence, the protective effect of three scFOS (1-Kestose, Nystose, and 1F-Fructofuranosylnystose) against ethanol-induced injury in gastric epithelial (GES-1) cells, and the underlying molecular mechanism involved was investigated in this study. Treatment with 7% ethanol decreased the cell viability of GES-1 cells, resulting in oxidative stress and inflammation. However, pretreatment with scFOS exhibited significant improvements in cell viability, and mitigated oxidative stress and inflammation. scFOS markedly elevated the protein expression of Nrf2, HO-1, SOD1 and SOD2, while suppressing the expression of Keap1. scFOS pretreatment could also maintain mitochondrial membrane potential balance and reduce apoptosis. In addition, scFOS was observed to reduce the protein level of NLRP3, Caspase-1 and ASC. In conclusion, scFOS served a preventive function in mitigating oxidative stress and inflammation in ethanol-exposed GES-1 cells through modulation of the Keap1/Nrf2 and NLRP3 inflammasome signaling pathways. Collectively, the results indicated that scFOS could significantly mitigate ethanol-induced gastric cell damage, suggesting its potential for safeguarding gastrointestinal health.

## Introduction

Alcohol ranks seventh among the global risk factors for death, causing a large number of deaths each year (1). Statistics as of 2020 showed that there were more than 740,000 cases of cancer-related to alcohol consumption globally, 76.7% of which were in men (2). Long-term excessive alcohol consumption results in harm to the gastric mucosa, which can develop a range of gastrointestinal tract diseases, including gastritis, peptic ulcers and gastric cancer (3). The mucosa serves as the primary barrier against pathogens and ensures the stability of the internal environment. The gastrointestinal mucosa plays a crucial physiological role as the biggest mucosal barrier organ in the body. Elevated ethanol concentrations directly erode the tissue of the gastric mucosa (4), causing gastric mucosal cell detachment, bleeding, and decreased mucus levels (5), resulting in gastric mucosal damage and consequent onset of disease. The pathophysiology of ethanol-mediated stomach damage involves inflammatory mediators and reactive oxygen species (ROS) generated by ethanol, which further promotes lipid peroxidation of cell membranes which in turn causes cell death and epithelial damage (6). Studies on gastric mucosal cells have shown that exposure to ethanol causes an increase in malondialdehyde (MDA) and nitric oxide (NO) production, while simultaneously reducing the production of important antioxidants such as superoxide dismutase (SOD), glutathione (GSH), and other enzymes (7, 8). This interplay causes an amplified state of oxidative damage, ultimately culminating in gastric mucosal lesions, accompanied by an acute inflammatory response and accelerates the release of pro-inflammatory inflammatory factors including tumor necrosis factor-alpha (TNF-α), interleukin-1 beta (IL-1β) and interleukin 6 (IL-6) (9–11).

Nuclear factor erythroid-2 related factor 2 (Nrf2) is a crucial transcription factor that regulates intracellular redox balance and cellular oxidative stress (12). Previous studies have confirmed that alcohol treatment increased ROS content and induced oxidative stress in GES-1 cells, which might activate the Nrf2 pathway and cause cell damage (13). Kelch-like ECH associated protein 1 (Keap1) controls Nrf2 activation negatively affecting the nuclear translocation of Nrf2 under unstressed conditions. Nrf2 increases the expression of downstream antioxidants via modulating the transcript and protein expression levels of heme oxygenase 1 (HO-1). Moreover, NOD-like receptor thermal protein domain associated protein 3 (NLRP3) inflammasome participates in the inflammatory injury of gastric mucosal (14).

Numerous studies have shown that many plant-derived polysaccharides exhibit excellent gastroprotective activity *in vivo* and *in vitro* (15, 16). Inulin is a linear fructan that consists of fructose molecules linked by β-2,1 glycosidic bonds, usually terminated by a glucose molecule (17), and its structural general formula is written as GFn. Inulin is naturally present in various eatable plants, among which chicory root has a high content and is considered to be the main natural raw material (18). The degree of polymerization (DP) of fructose molecules in inulin is usually between 2 and 60 (19). Due to its special glycosidic bond structure cannot be digested and broken down by human enzymes (20), almost 90% of inulin will enter the colon and be digested by the intestinal flora (21). Fructan with a degree of polymerization between 2 and10 is called fructo-oligosaccharides (FOS) (22). It has been demonstrated that the addition of inulin to the diet prevented lipid peroxidation in the stomach (23). The dietary addition of inulin or FOS contributes to the suppression of oxidative stress, which may prevent the onset of oxidative stress-related inflammatory responses (24, 25). Since oxidative stress and inflammation are important factors in the development of disorders affecting the digestive system, inulin and FOS with antioxidant and anti-inflammatory activities may contribute to the prevention and treatment of gastrointestinal (GI) diseases (26). It has also been noted that the antioxidant capacity of linear fructose seems to be correlated with its DP (27). The present study aimed to evaluate the protective ability and mechanism of three short-chain fructo-oligosaccharides (scFOS, DP3-5) in FOS, namely 1-Kestose, Nystose and 1^F^-Fructofuranosylnystose, against ethanol-induced gastric epithelial (GES-1) cells injury model.

## Materials and methods

### Chemical reagents

scFOS including 1-Kestose, Nystose and 1^F^-Fructofuranosylnystose were obtained from FUJIFLM Wako Pure Chemical Corporation (Osaka, Japan). The purity of scFOS measured by high performance liquid chromatography (Wakoosil 5NH_2_, Shimadzu LC-20, Japan) was ≥99%.

### Cell culture and treatment

GES-1 cell was obtained from the American Type Culture Collection (ATCC). Cells were cultured in HyClone RPMI medium with 10% fetal bovine serum (FBS, PAN-Seratech, Heilbronn, Germany) and 0.5% penicillin/streptomycin solution (Gibco, NY, United States) in a humidified incubator (Thermo Fisher Scientific, Langenselbold, Germany) at 37°C, 5% CO_2_. GES-1 cells were seeded in 6-well plates, 96-well plates or confocal microplates at a density of 1.5 × 10^5^ cells/mL for the subsequent treatment. After being treated, GES-1 cells were lysed with RIPA Lysis Buffer (Beyotime, Beijing, China) to collect cell lysates for subsequent experimental assays.

To explore the method of establishing ethanol-induced GES-1 cell damage model, cells were exposed to ethanol at various concentrations: 5, 6, 7, 8, and 9% for 2 h to determine cell viability. The ethanol concentration corresponding to a cell viability of approximately 60% was selected as the optimal modeling concentration. To determine the administration concentration of scFOS on GES-1 cells, cells were exposed to 1-Kestose, Nystose and 1^F^-Fructofuranosylnystose (dissolved in PBS) at various concentrations: 25, 50, 100 and 200 μg/mL for 24 h, following cell viability measurement. GES-1 cells were pretreated with scFOS at the selected concertation for 24 h, following ethanol exposure at the selected concertation for 2 h. The ameliorative effects of scFOS on cell viability, oxidative stress and inflammation were subsequently evaluated.

### Cell viability assay

Cell counting kit-8 assay kit (CCK-8, Beyotime, Beijing, China) was used to determine cell viability. GES-1 cells after treatment were incubated with100 μL of CCK-8 working solution (fresh medium and CCK-8 reagent were mixed in a 10:1 ratio) at 37°C and 5% CO_2_ for 60 min in the dark, the absorbance of each well was determined at 450 nm by a microplate reader (Spectra Max plus^384^; Molecular Devices, United States).

### Sod and MDA determination

The MDA content and SOD activity were determined separately using commercial kits according to the instructions (Beyotime, Beijing, China). The protein level of GES-1 cells was determined using KeyBio BCA Protein Assay Kit (KeyBionet, Nanjing, China) to normalize the MDA and SOD activity levels.

### Intracellular ROS generation assay

The intracellular ROS levels and superoxide anion (O^2−^) production were detected by using dichloro-fluorescein (DCFH-DA) and dihydroethidium (DHE) probes, respectively, (Beyotime, Beijing, China).

After being treated with scFOS and ethanol, GES-1 cells were exposed to 10 μM DCFH-DA or 5 μM DHE, and then incubated at 37°C under light protection for 30 min. The ROS levels were measured using a fluorescent enzyme labeling instrument and DHE fluorescent images were taken under a confocal microscope (Zeiss LSM 700, Carl Zeiss AG, Germany).

### Inflammatory cytokines assay

ELISA kits (Ruixin Biotechnology, Shanghai, China) were utilized to quantify the quantities of TNF-α, IL-1β and IL-6 in the cell lysates following the manufacturer’s instructions.

### NO production assay

Using a NO assay kit (Jiancheng Bioengineering, Nanjing, Jiangsu, China), NO concentration in cells is gauged by the transformation of NO in cell lysates into nitrate and nitrite with a NO assay kit. The absorbance of each well was measured at 550 nm using a microplate reader.

### Mitochondrial membrane potential assay

Mitochondrial Membrane Potential Assay Kit with JC-1 (Solarbio, Beijing, China) was used to measure changes in mitochondrial membrane potential caused by ethanol in GES-1 cells. JC-1 monomer fluoresces (green) were observed at excitation/emission (Ex/Em) 515 nm/529 nm, and JC-1 polymer fluoresces (red) were detected at excitation/emission (Ex/Em) 585/590. Changes in the potential of the mitochondrial membrane were detected by the shift in color of the fluorescence. Treated GES-1 cells in confocal dishes were incubated with JC-1 reagent (10 μM, 1 mL/well) for 20 min at 37°C protected from light and then imaged with confocal microscopy. The ratio of red to green fluorescence was utilized for determining changes in the mitochondrial membrane potential.

### TdT-mediated dUTP nick end labeling (TUNEL) assay

Apoptosis of GES-1 cells was assessed using the One Step TUNEL Apoptosis Assay Kit (Beyotime, Beijing, China). Briefly, cells were fixed with 4% paraformaldehyde (Beyotime, Beijing, China) for 30 min and then incubated with Terminal Deoxynucleotidyl Transferase containing fluorescein-dUTP for 60 min at 37°C. Finally, an Antifade Mounting Medium (Beyotime, Beijing, China) was added and then visualized by confocal microscopy.

### Immunofluorescence

GES-1 cells were treated and immobilized with 4% paraformaldehyde for 30 min, then blocked with 5% skim milk powder (Wako Pure Chemical, Osaka, Japan) for 2 h. Cells were incubated with anti-Nrf2 antibody (16396-1-AP, 1:1000; Proteintech, Chicago, IL, United States) at 4°C overnight. The next day, they were incubated with a fluorescent secondary antibody for 1 h. Antifade Mounting Medium with DAPI (Beyotime, Beijing, China) was added and images were taken with confocal microscopy.

### Western blot

The protein concentration in the cell lysate was quantified using KeyBio BCA Protein Assay Kit (Beyotime, Beijing, China). Following thorough mixing with loading buffer, lysates were boiled for 5 min. Following a 10% SDS-PAGE separation, the proteins were electrophoresed at 300 mA for 1.5 h, and subsequently transferred to a PVDF membrane (Millipore, MA, United States). The PVDF membranes were blocked with 5% skim milk powder for 2 h at room temperature and then co-cultured with the primary antibody overnight at 4°C. The next day, the PVDF membranes were co-incubated with secondary antibody for 1.5 h at room temperature. Finally, the membranes were immersed in ECL chemiluminescent solution, detected by Tanon Imager 4,600 system (Tanon, Shanghai, China) and analyzed by Image J software (NIH, Bethesda, MD, United States).

### Statistical analysis

The experimental data were analyzed by Graph Pad Prism version 8.0 (Graph Pad Software, San Diego, CA, United States). Results were expressed as means ± SEM, and the differences were analyzed by One-way ANOVA with Dunnett’s multiple comparison test, where *p* < 0.05 was considered statistically significant.

## Results

### Effect of different concentrations of scFOS and ethanol on the survival rate of GES-1 cells

**Figure 1:**
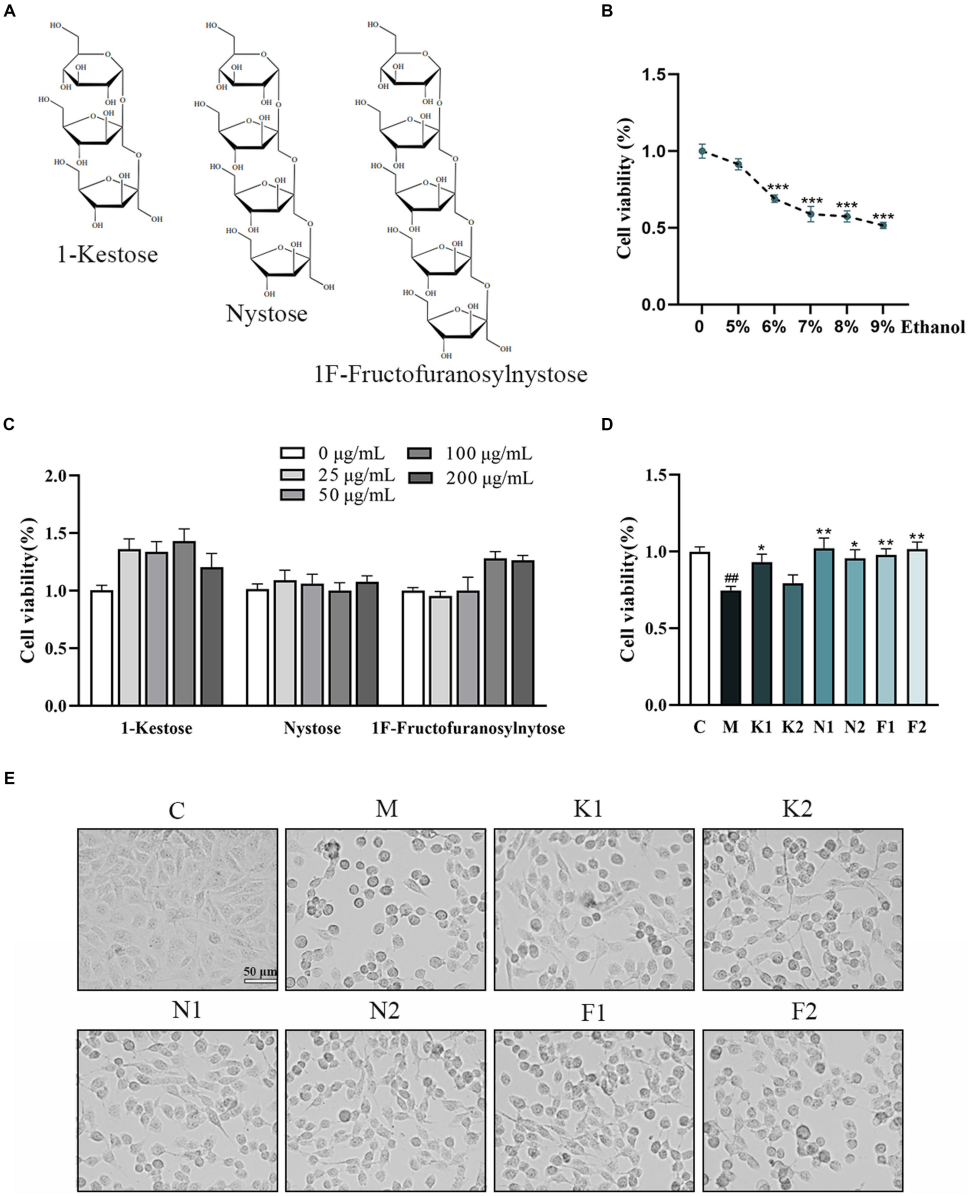
Effect of different concentrations of scFOS and ethanol on the survival rate of GES-1 cells. **(A)** The chemical structure of 1-Kestose, Nystose and 1^F^-Fructofuranosylnytose. **(B)** Concentration effect of ethanol on cell viability. **(C)** Concentration effect of 1-Kestose, Nystose, 1^F^-Fructofuranosylnytose on cell viability. **(D)** Cell viability was measured after pretreatment with 50, 100 μg/mL 1-Kestose (K1, K2), Nystose (N1, N2) and 1^F^-Fructofuranosylnytose (F1, F2) for 24 h and incubation with 7% ethanol for 2 h. **(E)** GES-1 cell morphology under the condition of panel **(D)** treatment. The cell viability of each group was expressed in percentage in comparison with the C group. *p < 0.05, **p < 0.01 or ***p < 0.001 compared with M group, ^**#**^*p* < 0.05, ^**##**^*p* < 0.01, ^**###**^*p* < 0.001 compared with the C group.

The chemical structures of the three scFOS were shown in Figure 1A. Figure 1B showed that the viability of GES-1 cells decreased with the increase of ethanol concentration from 5 to 9% with incubation for 2 h. According to our results and literature search, we selected 7% ethanol with an inhibition rate of about 40% as (Model, M) concentration for our further study (28).

The cell viability of GES-1 cells treated with different concentrations of scFOS for 24 h was shown in Figure 1C. scFOS at the concentration from 0 mg/mL (Control, C) to 200 mg/mL for 24 h did not affect GES-1 cells viability. scFOS at concentrations of 50 and 100 μg/mL were selected for subsequent experiments.

As shown in Figure 1D, GES-1 cells treated with the three scFOS at 50 μg/mL and 100 μg/mL for 24 h markedly improved cell viability compared to that in the M group. After the aforesaid ethanol treatment, the cells were found to be shrunken and rounded. However, the morphology of cells pretreated with scFOS was significantly improved (Figure 1E). These results indicated that scFOS improved cell viability and morphology in ethanol-exposed GES-1 cells.

### scFOS ameliorated oxidative stress in ethanol-treated GES-1 cells

**Figure 2:**
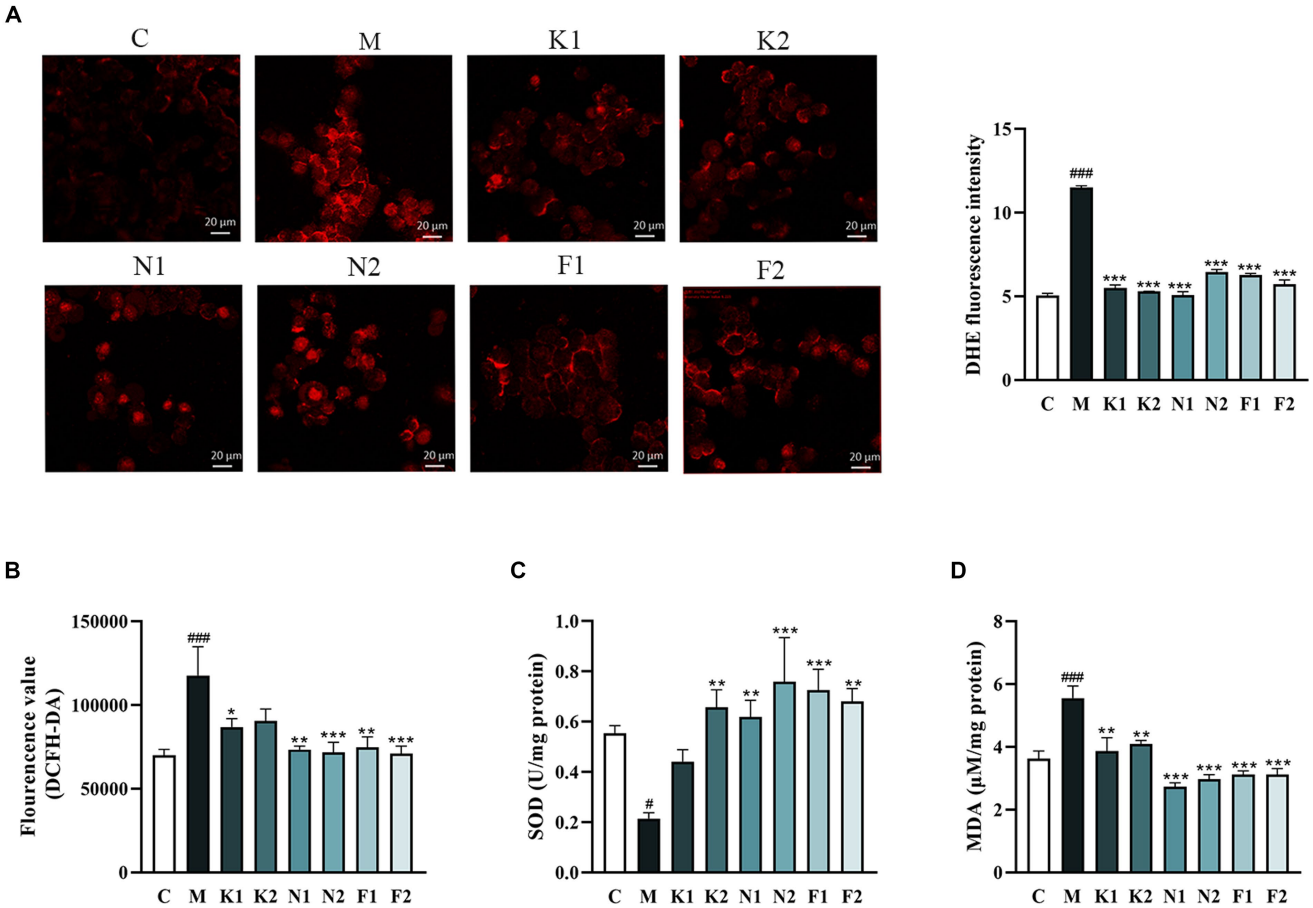
Effect of scFOS on oxidative stress in ethanol-treated GES-1 cells. **(A)** Intracellular superoxide anion (O^2−^) measured by DHE staining and the fluorescence intensity analysis. **(B)** Intracellular ROS measured by DCFH-DA fluorescent probe. MDA content **(C)** and SOD activity **(D)** were measured by biochemical kits. *p < 0.05, **p < 0.01 or ***p < 0.001 compared with M group, ^**#**^*p* < 0.05, ^**##**^*p* < 0.01, ^**###**^*p* < 0.001 compared with C group.

The O^2−^production in GES-1 cells detected using DHE staining was shown in Figure 2A. O^2−^ over-production was observed in GES-1 cells under 7% ethanol, and significantly reversed under scFOS administration. The DCFH-DA staining showed that ethanol exposure promoted intracellular ROS production, which was attenuated after scFOS treatment (Figure 2B). As shown in Figure 2C, the SOD activity of the M group was significantly reduced, while scFOS treatment significantly elevated SOD activity. Meanwhile, Nystose (N1, N2) and 1^F^-Fructofuranosylnystose (F1, F2) pretreatment exhibited better enhancement of SOD activity in ethanol-damaged GES-1 cells. As shown in Figure 2D, scFOS pretreatment significantly decreased the MDA content in comparison to that in M group. Among them, Nystose (N1, N2) and 1^F^-Fructofuranosylnystose (F1, F2) pretreatment could better reduce the MDA content in ethanol-exposed GES-1 cells. These findings suggested that pretreated with scFOS could attenuate ethanol-induced oxidative stress by suppressing ROS production and lipid peroxidation, as well as increasing intracellular SOD activity, of which Nystose and 1^F^-Fructofuranosylnystose showed a better improvement effect.

### scFOS reduced oxidative damage via the Nrf2/Keap1 pathway

**Figure 3:**
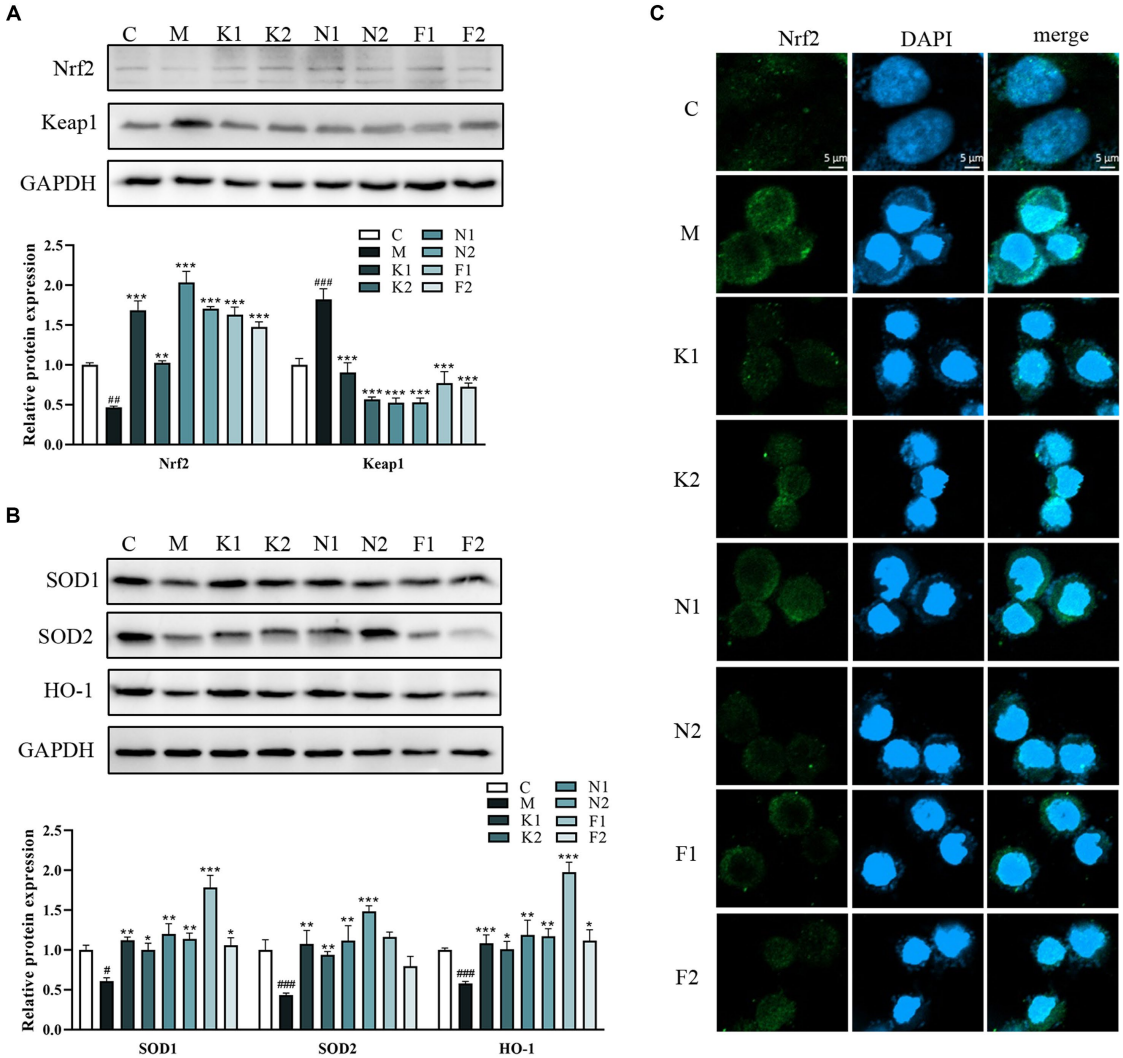
scFOS reduced oxidative damage via the Nrf2/Keap1 pathway. **(A)** Western blot analysis of Nrf2 and Keap1 expression. **(B)** Western blot analysis of SOD1, SOD2 and HO-1 expression. **(C)** Effect of ethanol stimulation on nuclear translocation of Nrf2 in GES-1 cells: Nrf2 (green) and DPAI (blue) in treated GES-1 cells were immunostained. Representative images of double staining are shown. Scale bar = 5 μm. *p < 0.05, **p < 0.01 or ***p < 0.001 compared with M group, ^**#**^*p* < 0.05, ^**##**^*p* < 0.01, ^**###**^*p* < 0.001 compared with C group.

Figure 3A showed that the Nrf2 expression level was upregulated and Keap1 expression was downregulated in the scFOS group in comparison with the M group. As shown in Figure 3B, compared with the C group, the expression of HO-1, SOD1 and SOD2 proteins in the M group was significantly decreased. Compared with the M group, HO-1, SOD1 and SOD2 protein expression of the scFOS administration group was significantly reduced to a level close to the normal group. Indeed, Nrf2 immunofluorescence also revealed an accumulation of nuclear Nrf2 in the scFOS treatment group, while Nrf2 expression in the cytoplasm was significantly reduced in comparison with those in the M group (Figure 3C). In addition, nuclear morphology was observed using DAPI staining, while cells with nuclear fragmentation were considered to be a marker of apoptosis (29). Compared to the C group, the nucleus of the ethanol-treated group was fragmented and the morphology was abnormal, which was improved after scFOS pretreatment. Therefore, scFOS could trigger the degradation of Keap1 and promote the entry of Nrf2 into the nucleus, thereby increasing the expression of antioxidants such as HO-1 and SOD to reduce the level of oxidative stress in cells. These results demonstrated that scFOS could improve ethanol-induced oxidative stress in GES-1 cells by regulating the Nrf2/Keap1 signaling pathway.

### scFOS inhibited inflammatory response and apoptosis by NLRP3/ASC/Caspase-1 pathway

**Figure 4:**
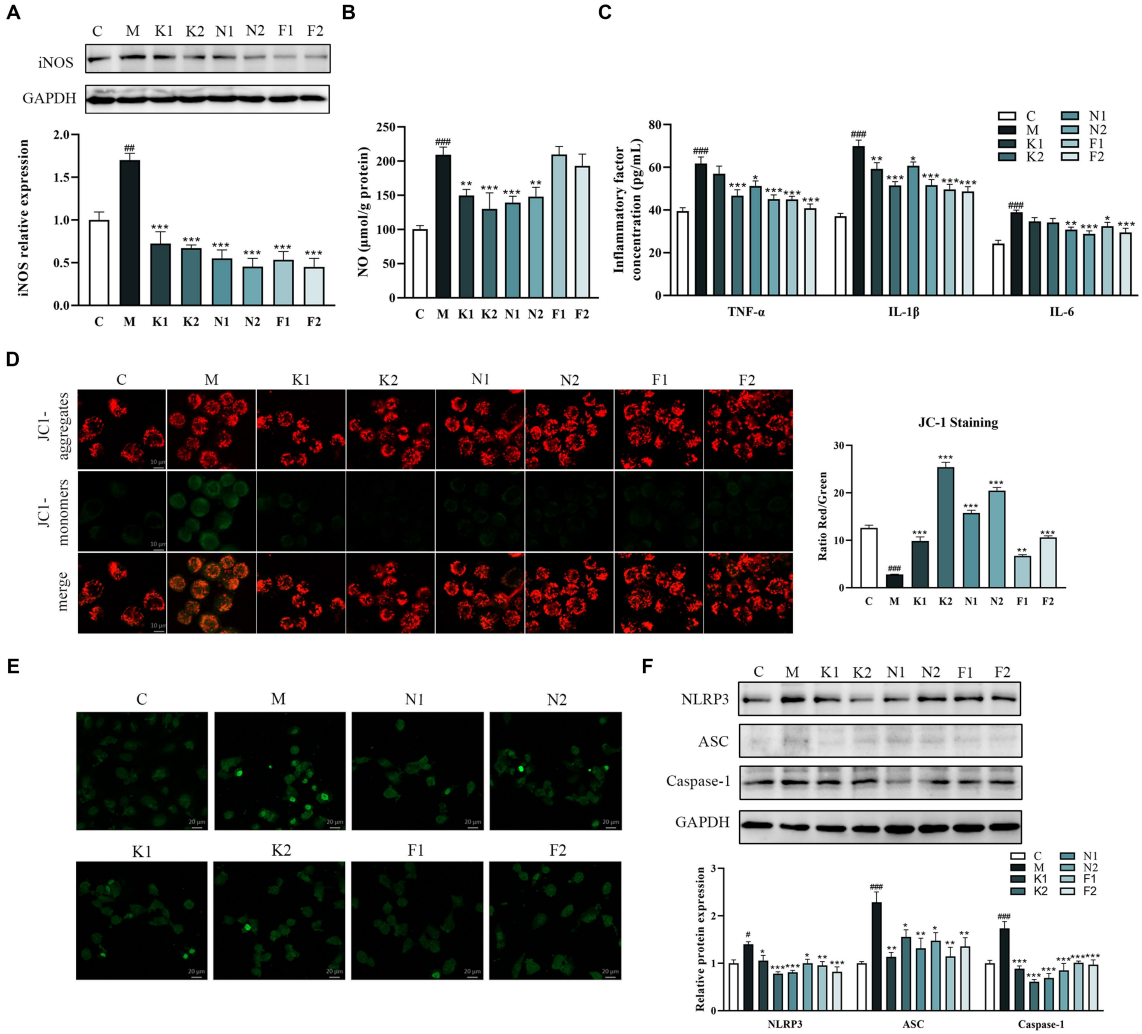
scFOS inhibited inflammatory response and apoptosis by NLRP3/ASC/Caspase-1 pathway. **(A)** Western blot analysis of iNOS expression. **(B)** NO production was evaluated by spectrophotometry. **(C)** Anti-inflammatory inflammatory factors TNF-α, IL-1β and IL-6 quantified by ELISA. **(D)** Quantitative evaluation of the MMP content in ethanol-treated GES-1 cells using the JC-1 polymer/monomer fluorescence ratio. **(E)** The levels of apoptosis were analyzed using a TUNEL detection kit. **(F)** Expression of NLRP3, ASC and Caspase-1 was examined using Western blot. *p < 0.05, **p < 0.01 or ***p < 0.001 compared with M group, ^**#**^*p* < 0.05, ^**##**^*p* < 0.01, ^**###**^*p* < 0.001 compared with C group.

The production of NO can be used as a predictor of the inflammatory effects in the disease process. As shown in Figures 4A,B, ethanol significantly increased NO and iNOS production in GES-1 cells in comparison to the C group. In the scFOS treatment group, all three scFOS could correct the significant decrease in iNOS content induced by ethanol. However, 1-Kestose and Nystose administration significantly reduced NO production in comparison with the ethanol-treated group, while there was no significant change in the 1^F^-Fructofuranosylnystose-treated group, indicating the potential of scFOS to suppress the inflammatory response.

Proinflammatory cytokines, including TNF-α, IL-1β and IL-6, are crucial mediators in the majority of inflammatory pathologies. Therefore, we further determined the effect of scFOS on the release of TNF-α, IL-1β and IL-6 in ethanol-exposed GES-1 cells. As demonstrated in Figure 4C, ethanol treatment considerably increased TNF-α, IL-1β and IL-6 levels in comparison to the C group, while scFOS administration dramatically decreased these inflammatory factor levels. The above results suggested that scFOS may exert excellent anti-inflammatory effects in ethanol-treated GES-1 cells.

Apoptosis can be detected early by observing the change in JC-1’s fluorescence from red to green. The red/green fluorescence ratio of the M group was considerably reduced following ethanol treatment for 2 hours, as seen in Figure 4D. All three scFOS significantly increased the red/green fluorescence ratio in comparison with that in the ethanol-treated GES-1 cells, with 1-Kestose and Nystose exhibiting a better improvement effect than 1^F^-Fructofuranosylnystose. JC-1 staining results showed that all scFOS treatment groups could improve ethanol-induced apoptosis in a dose-dependent manner, which was further confirmed by TUNEL analysis. As shown in Figure 4E, the positive staining cells in the M group were significantly elevated than those in the C group and scFOS treatment group.

The NLRP3 signaling pathway is crucial for both apoptosis and the inflammatory response. Western blot analysis showed that ethanol induction increased the expression of NLRP3, apoptosis-associated speck-like adaptor protein (ASC) and cysteinyl aspartate specific proteinase 1 (Caspase-1) (Figure 4F). However, pretreatment with three scFOS significantly inhibited ethanol-induced activation of these proteins, suggesting that scFOS can also prevent ethanol-induced inflammatory response and apoptosis by inhibiting NLRP3 signaling.

## Discussion

Inulin is a water-soluble storage polysaccharide, which belongs to a class of non-digestible carbohydrates. With its function of improving health and regulating the gastrointestinal system, inulin is widely used in the development of functional foods (30). Fructans with a degree of polymerization between 3 and 5 are called scFOS. It has been reported that the degree of polymerization of scFOS may affect its biological activity (31). Related studies have shown that 1^F^-Fructofuranosylnystose could scavenge free radicals *in vitro*, but its antioxidant mechanism is not yet clear. We studied the protective effect and mechanism of scFOS in FOS, 1-Kestose, Nystose and 1^F^-Fructofuranosylnystose, on ethanol-induced GES-1 cell injury.

In cell experiments, GES-1 cells were treated with different concentrations of ethanol to establish an alcohol injury model. Through the inverted microscope observation, it was found that the cells treated with a high concentration of ethanol would cause the cells to contract and round, and then fall off from the bottle wall (32). The same phenomenon was also confirmed in the present study. Direct observation of cell status is helpful for preliminary judgment of screening concentration. A study demonstrated that the cell survival rate was approximately 50% after exposure to 3% ethanol for a duration of 4 h (33). Furthermore, the study outlined the recommended concentration (2–8%) and duration (2–6 h) to minimize volatilization in ethanol molding (33). In this study, ethanol treatment for 2 h and the cell survival rate of about 60% was used as modeling criteria to explore the optimal concentration. It was found that 7% ethanol could meet the modeling requirements for 2 h. Ethanol intake can cause oxidative stress in cells, which damages GES-1 cells by increasing lipid peroxidation and decreasing the activity of antioxidant enzymes (34).

In the development of ethanol-induced gastric mucosal injury through oxidative stress, reactive oxygen species (ROS) play a crucial role (35). Therefore, the evaluation of the resistance and repair ability of GES-1 cells to alcohol injury can be indicated by their antioxidant activity. As a class of enzymes that catalyze the disproportionation of superoxide anion radicals to H_2_O_2_ and O_2_, SOD plays a crucial role in helping the body against oxidative damage (36). Ethanol treatment induced oxidative stress, increased intracellular ROS and MDA content, and decreased SOD activity (37). These findings are consistent with the results of our study, which demonstrated that ethanol caused a significant increase in ROS levels in GES-1 cells and disrupted the antioxidant system, as represented by SOD. scFOS pretreatment could protect GES-1 cells from oxidative stress, and the antioxidant effects of Nystose and 1^F^-Fructofuranosylnystose are better than that of 1-Kestose.

ROS generation is regulated by Nrf2 signaling (38). Under normal conditions, Nrf2 and Keap1 are easily degraded in the cytoplasmic binding (39, 40). In oxidative stress situations, Keap1 undergoes a conformational shift or Nrf2 is directly phosphorylated in response to internal and external free radicals and substances stimulating the cell (41). Nrf2 in cells can get rid of the inhibition of Keap1 and accumulate in the nucleus through the nuclear translocation signal of Neh1 domain on Nrf2 (42). Upon entering the nucleus, activated Nrf2 triggers the production of HO-1 and downstream antioxidant proteases (43, 44). The up-regulation of HO-1 may be one of the most important cell protection mechanisms in the event of cell stress (45). The possible mechanism of the Nrf2/HO-1 signaling pathway in the protective effect of scFOS on alcohol-injured GES-1 cells was then investigated. Our results showed the suppression of Keap1 and the promotion of Nrf2, HO-1, SOD1 and SOD2 by scFOS in the stimulation of ethanol, confirming that scFOS could prevent ethanol-induced GES-1 cell damage by alleviating oxidative stress.

Inflammation, as a biological response to potential harm, stands out as a pivotal process in the gastric mucosal defense mechanism (46). Ethanol promotes inflammation, which in turn leads to the accumulation of inflammatory cytokines. NLRP3 inflammasome plays a key role in many diseases, and it may establish a bridge between inflammation and oxidative stress (47, 48). NLRP3 inflammasome is an intracellular complex associated with inflammatory response, which induces the production of mature IL-1β and triggers apoptosis through caspase-1 cleavage, playing an important role in the formation of gastric ulcer (49–51). The marker of early apoptosis is the damage of active mitochondria, which is closely related to cellular oxidative damage (52). In this study, the JC-1 probe was used to observe the decrease of MMP in cells under confocal microscopy. DAPI staining and TUNEL staining provided evidence for ethanol-induced apoptosis, which was characterized by nuclear condensation and fragmentation. Our study found that scFOS could significantly inhibit the increase of ethanol-induced NLRP3, ASC and Caspase-1 protein expression in GES-1 cells, indicating that scFOS enhanced ethanol-induced NLRP3 inflammasome activation-mediated apoptosis, suggesting that scFOS may protect GES-1 cells from ethanol-induced damage by inhibiting NLRP3. At the same time, we also detected that the levels of IL-1β, TNF-α, IL-6, iNOS and NO in alcohol-induced GES-1 cells were significantly increased, and scFOS pretreatments could reverse this phenomenon to varying degrees. Interestingly, we observed that 1^F^-Fructofuranosylnystose could not significantly reduce the expression of NO in cells, but the expression level of iNOS in cells decreased significantly, which may be that 1^F^-Fructofuranosylnystose could not inhibit the non-enzymatic NO pathway in cells (53).

## Conclusion

In summary, this study showed that scFOS could protect GES-1 cells from ethanal-induced oxidative stress and inflammatory response via Nrf2 and NLRP3 inflammasome-related pathway proteins. These findings demonstrated that scFOS could successfully prevent ethanol-induced stomach cell injury *in vitro*, suggesting its potential for safeguarding gastrointestinal health. However, further research is needed to fully elucidate the extent of scFOS’s benefits in addressing stomach diseases.

## Data availability statement

The raw data supporting the conclusions of this article will be made available by the authors, without undue reservation.

## Author contributions

YC: Writing – review & editing, Writing – original draft, Visualization, Methodology, Investigation. YZ: Writing – review & editing, Visualization, Methodology, Formal analysis. HLu: Methodology, Formal analysis, Writing – review & editing. WZ: Visualization, Methodology, Investigation, Writing – review & editing. YG: Formal analysis, Data curation, Writing – review & editing. GN: Methodology, Formal analysis, Writing – review & editing. XM: Visualization, Methodology, Data curation, Writing – review & editing. HLv: Visualization, Methodology, Investigation, Writing – review & editing. XQ: Resources, Writing – review & editing. XD: Writing – review & editing, Writing – original draft, Visualization, Funding acquisition, Data curation, Conceptualization. JC: Writing – review & editing, Visualization, Resources, Project administration, Funding acquisition, Conceptualization.
